# Non-Classic (Surrepticius) Scabies Presenting as Erythematous Painful Palmar Pustules in an Octagenarian

**DOI:** 10.7759/cureus.9542

**Published:** 2020-08-03

**Authors:** Philip R Cohen, Christopher Crowley, Christof Erickson, Antoanella Calame

**Affiliations:** 1 Dermatology, San Diego Family Dermatology, National City, USA; 2 Dermatology, Compass Dermatopathology, San Diego, USA; 3 Dermatology/Dermatopathology, Compass Dermatopathology, San Diego, USA; 4 Dermatology, Scripps Memorial Hospital, La Jolla, USA

**Keywords:** atypical, burrow, mite, palmar, pustule, pustulosis, sarcopetes, scabiei, scabies, surrepticius

## Abstract

Sarcoptes scabiei classically presents with pruritic burrows. In addition to finger and toe web lesions, the penis and scrotum of men, the breast and areola of women, and the buttocks and axillae of both gender are common locations for mite-associated lesions. Scabies surrepticius refers to mite-related lesions that are not classical in morphology and/or distribution; there are several subtypes of non-classic or atypical scabies. Scabies surrepticius not only occurs in immunosuppressed individuals, but also in infants and children, and in elderly patients. An elderly woman developed mite-related bilateral erythematous painful palmar pustules that were clinically suspicious for a primary neutrophilic dermatosis; she also had a concurrent bacterial infection. The diagnosis of surrepticius scabies was established after evaluation of the deeper levels of her tissue biopsy specimen demonstrated a mite in the epidermis. All of her symptoms and lesions resolved after treatment with oral and topical antiscabetic therapy, and systemic antibiotics. Since the clinical presentation of the mite-related lesions can mimic those of other dermatologic or systemic conditions, a high degree of suspicion for the diagnosis of mite infestation must be entertained. Therefore, the clinician should consider the possibility of scabies surrepticius in any patient who develops new cutaneous lesions that are not typical for a defined dermatosis or do not resolve or improve after treatment.

## Introduction

Scabies infestations classically present with *Sarcoptes scabiei *mite-related skin manifestations. These include burrows that are often located on the finger and toe web spaces, wrists and dorsal hands, and lower legs and feet. In addition, cutaneous scabies-associated lesions can characteristically appear on men’s scrotum and penis, women’s areola and breast, and the axillae and buttock of both men and women [1].

However, an atypical presentation of scabies can have a non-classic appearance. The mite infestation in these individuals is referred to as scabies surrepticius. The morphology and distribution of the mite lesions in patients with this variant of scabies infestation can masquerade as other skin or systemic conditions [2-5].

The features of an elderly woman who experienced a unique presentation of scabies surrepticius manifesting as mite-associated bilateral erythematous painful palmar pustules that were clinically suspicious for a primary neutrophilic dermatosis are described. She also had a concurrent bacterial infection that altered the appearance of her scabies infestation. The possibility of non-classic (surrepticius) scabies should be considered in individuals with acquired skin lesions that are not characteristic for a specific dermatosis or do not appropriately respond to therapeutic intervention.

## Case presentation

An afebrile 86-year-old woman presented for evaluation of new tender lesions on both of her hands. Four days earlier, she had noticed asymptomatic blisters on her left palm. The blisters subsequently developed into painful pustules and similar lesions were appearing on her right palm. Her past medical history was significant for diabetes mellitus and hypertension; she had not started any new medications.

**Figure 1:**
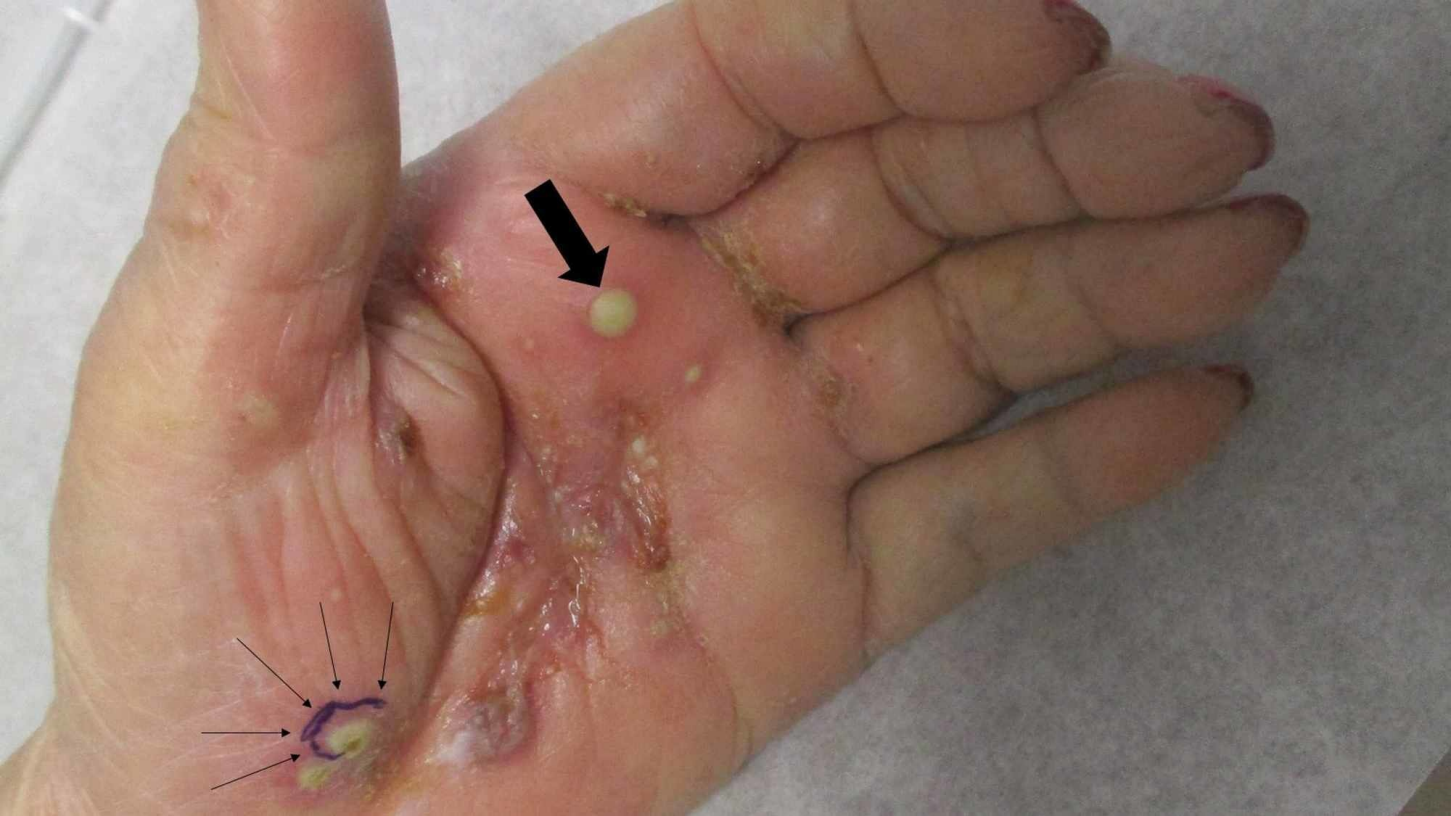
Scabies surrepticius presenting as red tender pustules on the left palm of an 86-year-old woman The hand is erythematous and swollen; these clinical features correspond to the culture-positive bacterial cellulitis from group A Streptococcus and methicillin-susceptible Staphylococcus aureus; the bacterial culture was performed from the distal pustule located closest to the second and third digits (single thick black arrow). The scabies-associated lesions include the painful palmar pustules and blisters; the biopsy site is circled in purple ink (and demarcated by multiple thin black arrows). The morphology of the scabies-associated palmar skin lesions mimics not only palmar pustulosis and pustular vasculitis, but also Sweet syndrome and its dorsal hand variant.

Cutaneous examination showed swollen hands with erythematous palms containing tender blisters in the central area and firm painful pustules distally and proximally (Figure 1). The remainder of her skin examination did not reveal any lesions. Specifically, her plantar feet were clear, she had no abnormalities of her nail plates, and there were no burrows on her fingers, finger webs, wrists, or other body sites.

The clinical differential included palmar pustulosis (which is a variant of psoriasis vulgaris), bacterial infection (cellulitis), acute febrile neutrophilic dermatosis (Sweet syndrome), neutrophilic dermatosis of the dorsal hands (which is a variant of Sweet syndrome and may have palmar lesions), and pustular vasculitis. She was empirically started on oral antibiotic, doxycycline monohydrate (100 milligrams twice daily), for 10 days. She was also treated (three times daily to the affected areas on her palms) with a high-potency topical corticosteroid: halobetasol 0.05% ointment. 

A culture for bacteria was performed from the distal pustule closest to her fingers. The bacterial culture grew both group A Streptococcus and methicillin-susceptible Staphylococcus aureus. Both organisms were susceptible to doxycycline.

**Figure 2:**
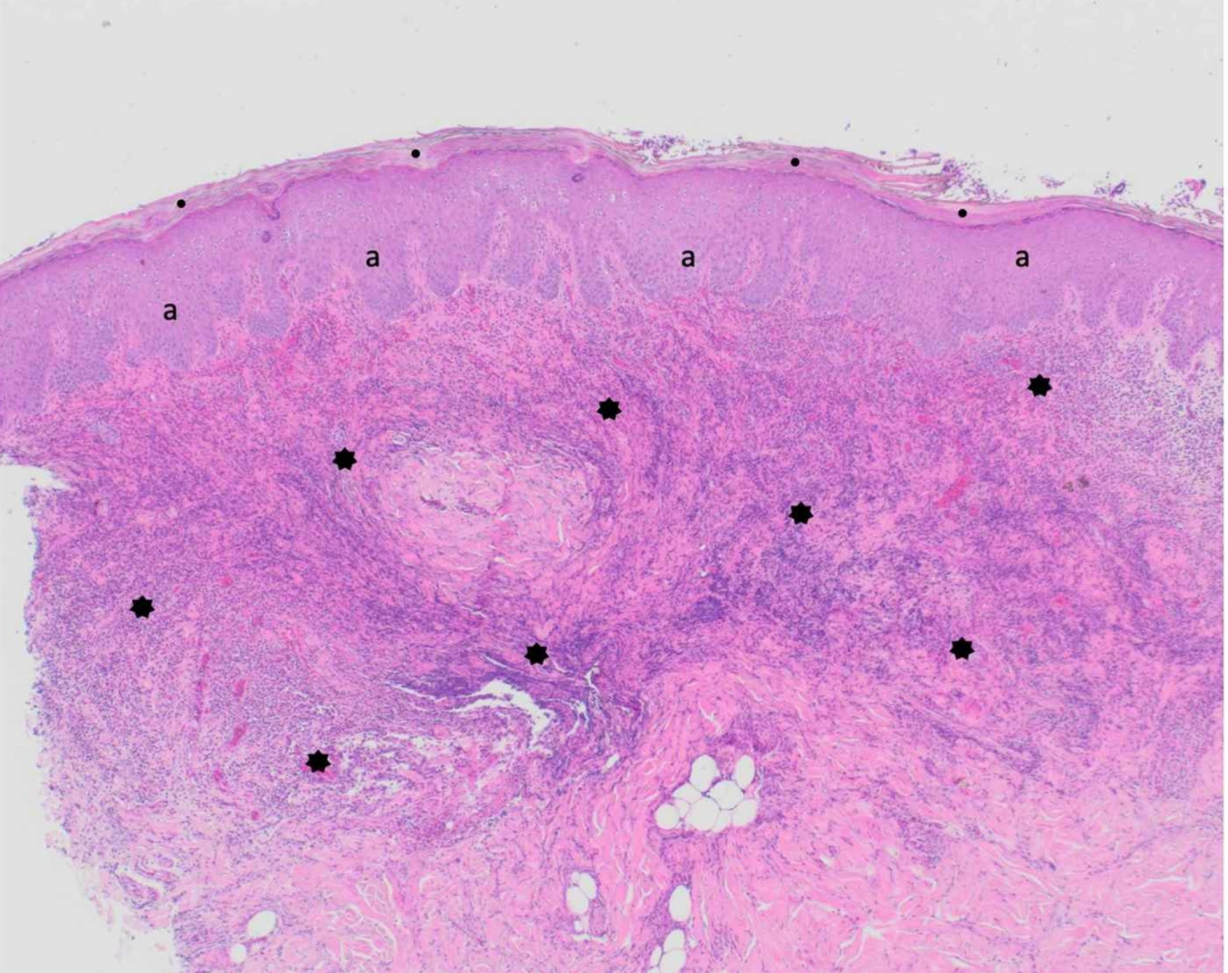
Microscopic examination of the initial section from the skin lesion biopsy of scabies surrepticius mimics a neutrophilic dermatosis The hematoxylin and eosin-stained tissue shows hyperkeratosis (thickening of the stratum corneum) (black circles), acanthosis (thickening of the remainder of the epidermis) (black a) with elongated rete ridges, and an intense diffuse neutrophilic infiltrate that not only fills the papillary dermis but also extends deeply into the reticular dermis (black stars). The pathologic differential includes abscess, acute febrile neutrophilic dermatosis (Sweet syndrome), pustular dermatosis of the dorsal hands, and pustular vasculitis (hematoxylin and eosin: x4).

A skin biopsy was also performed at the edge of the proximal pustule. Microscopic examination of the tissue specimen shows compact thickening of the stratum corneum without (orthokeratosis) and with (parakeratosis) retention of the nuclei, thickening of the epidermis (acanthosis) with elongation of the rete ridges, and a diffuse dense infiltrate of neutrophils that extends from the papillary dermis into the deep reticular dermis (Figure 2). Preliminary correlation of the clinical presentation and pathologic findings favored the diagnosis of a neutrophilic dermatosis, such as Sweet syndrome or neutrophilic dermatosis of the dorsal hands; however, the findings were also compatible with abscess secondary to a bacterial infection.

**Figure 3:**
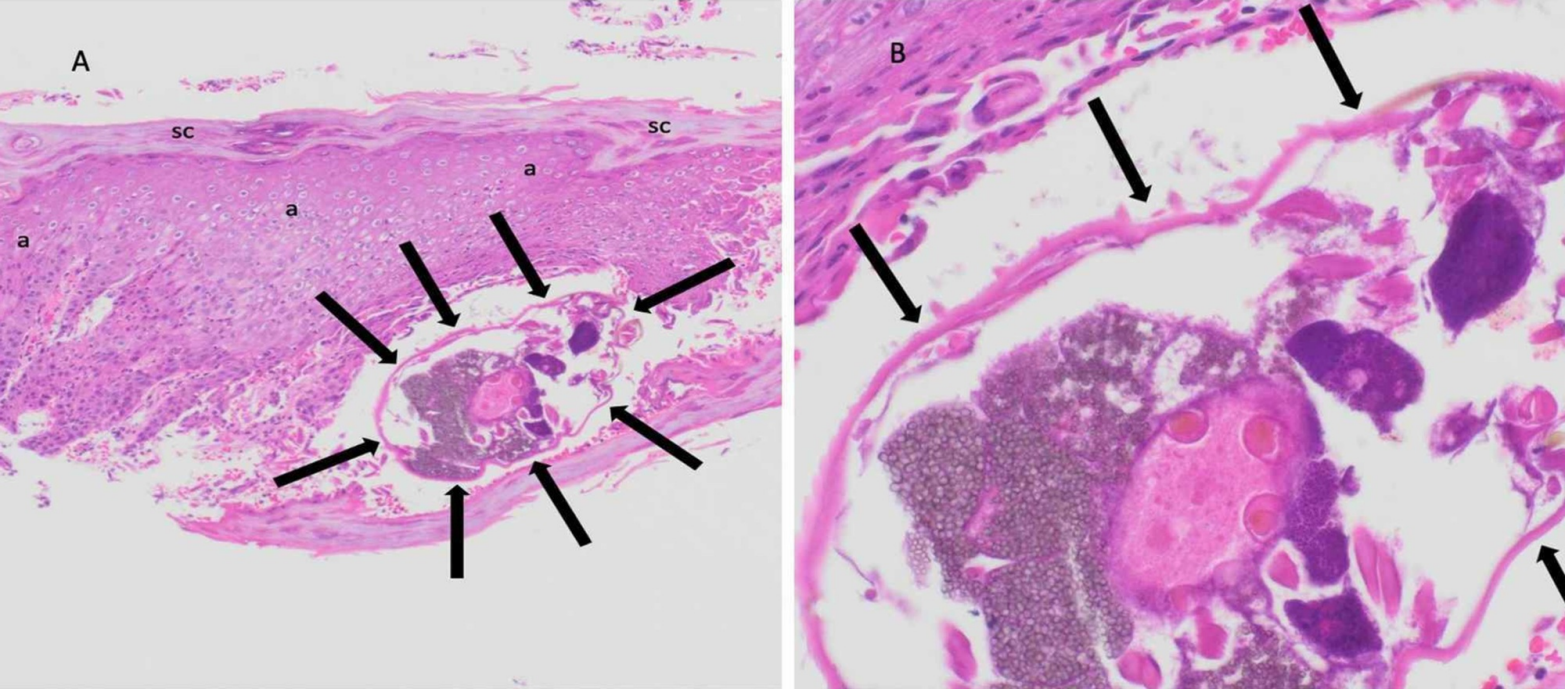
Microscopic examination of the deeper section from scabies surrepticius skin lesion biopsy demonstrates a mite in the epidermis Distant (A) and closer (B) views of the hematoxylin and eosin-stained tissue show a *Sarcoptes scabiei *mite (black arrows) beneath the stratum corneum (black sc) at the lateral side of the specimen; the location is the pustule edge and the top layers of the epidermis appears to be below the mite secondary to processing artifact. There is thickening (acanthosis) of the epidermis (black a) adjacent to the edge of the pustule (hematoxylin and eosin: a, x10; b, x20).

Deeper sections of the tissue specimen were performed to evaluate (using special stains) for infectious organisms. No bacteria, fungi, or mycobacteria were observed. However, a scabies mite was identified in the epidermis beneath the stratum corneum (Figure 3). Hence, subsequent correlation of the clinical features and pathology findings established the diagnosis of non-classic (surrepticius) scabies infestation presenting as palmar pustules.

The patient’s scabies infestation was treated not only topically, but also systemically. On days 1 and 8, she applied permethrin 5% cream from her neck to her toes overnight and received 12 milligrams of ivermectin. In addition, she completed all 10 days of oral antibiotic therapy and discontinued the topical corticosteroid ointment.

The biopsy-site sutures were removed after two weeks. At this follow-up appointment, the bilateral palmar swelling and tenderness had resolved. Also, all of the blisters and pustules had cleared.

## Discussion

Non-classic (surrepticius) scabies has several variants (Table 1) [2-5]. In children, it can masquerade as Langerhans cell histiocytosis and urticaria pigmentosa. Indeed, similar to our patient, scabies in infants can present with pustules on the palms and soles mimicking acropustulosis of infancy; however, to the best of our knowledge, we are not aware of this presentation occurring in adults [2].

**Table 1:** Subtype classification of scabies surrepticius by disease group or morphologic appearance ^a^These include acute febrile neutrophilic dermatosis (Sweet syndrome), neutrophilic dermatosis of the dorsal hands (variant of Sweet syndrome), and pustular vasculitis.

Disease group or morphology appearance	Scabies surrepticius subtype
Autoimmune disease	Systemic lupus erythematosus-like
Infiltrative disease	Langerhans cell histiocytosis-like, urticaria pigmentosa-like
Miscellaneous disease	Ecchymoses, hidden, scalp, urticaria
Nodules	Nodular, prurigo nodularis-like
Papulosquamous disease	Crusted, pityriasis rosea-like
Pustules	Acropustulosis of infancy-like, neutrophilic dermatosis-like^a^, palmoplantar pustulosis-like
Vesiculobullous disease	Bullous, dermatitis herpetiformis-like

Atypical scabies presentations are also more prevalent in individuals with congenital or iatrogenic immunodeficiency. For example, scabies lesions typically only occur below the neck; however, not only in immunosuppressed persons but also in infants and children, the mite infestation can be associated with scabies-related scalp lesions. Similarly, crusted scabies (previously referred to as Norwegian scabies) with keratotic plaques, each containing numerous mites, is a surrepticius scabies variant that more frequently occurs in the immunocompromised patient population [2,3].

Similar to our patient, scabies can present in the elderly. In this patient population, the bullous variant of scabies surrepticius may be diagnosed. Establishing the diagnosis can be challenging since the lesions mimicking bullous pemphigoid not only clinically, but also occasionally on light and immunofluorescence microscopy evaluation [2,6].

Bacterial organism may concurrently be present in scabies lesions. They can represent impetiginization of the skin secondary to excoriation of the lesions. Alternatively, similar to our patient, they can cause cellulitis and/or abscess [7,8].

We suspect that the palm swelling and painful erythema she experienced were secondary to erysipelas and/or impetigo from the bacterial organisms. Both bacteria were susceptible to the systemic antimicrobial agent. Her symptoms and findings completely cleared after antibiotic treatment.

The initial impression of our patient’s pustules was either palmoplantar pustulosis (a variant of psoriasis restricted to the palms and soles) or a primary neutrophilic dermatosis. The absence of an intraepidemal pustule on the biopsy tissue specimen excluded the former possibility. However, the intense infiltrate of neutrophils was consistent with Sweet syndrome or its dorsal hand variant [9,10].

The discovery of a scabies mite on deeper sections of the tissue block was unexpected. The location of the mite, beneath the stratum corneum of the pustule edge, and the absence of bacterial organisms suggest that the pustules were mite associated. Hence, we classified our patient’s mite infestation as surrepticius scabies. Her palmar pustules and blisters completely resolved after treatment with antiscabetic topical and oral agents.

## Conclusions

Scabies surrepticius refers to *Sarcoptes scabiei* mite infestations that present with lesions that do not have a classic morphology. Atypical scabies lesions occur in immunocompetent people; however, they more commonly present in immunosuppressed individuals, in infants and children, and in elderly patients. Our patient was elderly and her scabies surrepticius presented as erythematous painful palmar pustules that mimicked palmoplantar pustulosis and neutrophilic dermatosis. Her palmar symptoms, redness, and swelling may in part or completely be secondary to the concurrent presence of the cultured bacterial organisms that either impetiginized the scabies lesions or infected her hands. The acquisition of cutaneous lesions that are not typical for a defined dermatosis or do not resolve or improve after treatment should prompt the clinician to consider the possibility of scabies surrepticius.
